# Plasma creatine, estimated intramuscular creatine, transcellular gradient and the risk of mortality: Results from the PREVEND study

**DOI:** 10.1111/eci.70110

**Published:** 2025-08-21

**Authors:** Caecilia S. E. Doorenbos, Adrian Post, Mariken E. Stegmann, Casper F. M. Franssen, Robin P. F. Dullaart, Gerjan Navis, Margery A. Connelly, Stephan J. L. Bakker

**Affiliations:** ^1^ Department of Internal Medicine, University Medical Center Groningen University of Groningen Groningen The Netherlands; ^2^ Department of Primary and Long‐Term Care, University Medical Center Groningen University of Groningen Groningen The Netherlands; ^3^ Labcorp Morrisville North Carolina USA

**Keywords:** creatine, general population, mortality risk, muscle mass

## Abstract

**Background:**

Creatine, an endogenous compound essential for energy metabolism and cellular function, has been associated with numerous beneficial effects in sports and overall health. Here, we investigated relationships between plasma creatine concentration, estimated intramuscular creatine concentration, and all‐cause mortality in the general population.

**Methods:**

In a Dutch prospective population‐based cohort, plasma creatine concentration, 24‐h urinary creatinine excretion and muscle mass (assessed with bio‐electrical impedance analysis) were measured in 5127 participants. Total creatine pool size, calculated from 24‐h creatinine excretion (assuming a 1.7% daily excretion of the total creatine pool), was divided by muscle mass to estimate intramuscular creatine concentrations. Transcellular gradient was calculated as intramuscular concentration divided by plasma concentration. Hazard ratios for mortality per doubling were assessed using multivariable Cox proportional hazard models, adjusting for common cardiovascular risk factors for mortality.

**Results:**

Median plasma creatine concentrations were 41 [30–54] μmol/L in females and 28 [21–38] μmol/L in males. Mean intramuscular creatine concentrations were 30 ± 5.0 mmol/kg in females and 27.4 ± 5.0 mmol/kg in males. Median transcellular creatine gradients were 734 [550–1011] in females and 955 [715–1324] in males. Higher intramuscular creatine concentrations were associated with lower mortality in females (HR (95% CI) = .43 (.2; .66)); with a weaker trend in males (HR (95% CI) = .73 (.53; 1.02)). Plasma creatine concentrations were not associated with mortality.

**Conclusion:**

Higher estimated intramuscular creatine concentrations are strongly associated with lower all‐cause mortality in females, with a weaker trend in males. Future research should explore causality, as well as further explore the remarkable sex difference.

## Graphical abstract

Plasma creatine and intramuscular creatine concentrations were assessed using nuclear magnetic resonance and bio‐electrical impedance analysis in a general population‐based cohort (*n* = 5127). Cox‐regression analyses were performed to investigate associations with mortality. Higher intramuscular creatine concentrations were associated with lower mortality in females (HR (95% CI) = .43 (.20; .66)), with a weaker trend in males (HR (95% CI) = .73 (.53; 1.02)). Plasma creatine concentrations were not associated with mortality. Future research should explore causality and should further explore the remarkable sex difference.

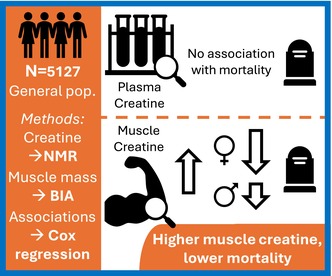

## 1 INTRODUCTION

Creatine is a prominent endogenous nitrogenous compound essential for energy metabolism and cellular function. Creatine in its phosphorylated form, phospho‐creatine, primarily serves as an immediately available energy reservoir in tissues with dynamic and high energy requirements, such as skeletal muscles, the brain and the heart. This energy buffering function is achieved via the creatine kinase system, which mediates the transfer of high‐energy phosphate groups from phosphocreatine to ADP, facilitating the rapid regeneration of ATP from ADP.^1^, ^2^ Besides acting as a buffer, the creatine kinase system also works as a continuous energy transport system between ATP production sites (mitochondria and glycolysis) and ATP consumption sites, for example, for muscle contraction and relaxation.^3^ Due to a continuous loss of the bodily creatine pool by spontaneous, non‐enzymatic conversion into creatinine, the creatine pool needs continuous replenishment through dietary intake and/or endogenous synthesis.^1^

Creatine has been associated with a wide range of health effects. Supplementation with creatine is linked to a myriad of beneficial outcomes, both in athletic performance and in general health, including enhanced exercise performance, increased muscle growth potential, accelerated recovery from exercise, improved cognitive function and improved cardiovascular markers such as cholesterol and triglycerides levels.^4^ On the other hand, although the exact underlying mechanisms have yet to be unravelled, several studies showed that higher plasma creatine concentrations are linked to adverse health outcomes. These include unfavourable metabolic and cardiovascular markers, such as elevated total cholesterol, lower HDL cholesterol and higher triglycerides, higher glucose and insulin levels and an increased incidence of diabetes and hypertension.^5^, ^6^ Notably, some of these associations appear to be stronger in males compared to females.^5^, ^6^

Given these contrasting associations of creatine‐related markers, we aimed to explore the relationship between plasma creatine concentrations and intracellular, specifically intramuscular creatine concentrations. Furthermore, we aimed to prospectively examine the associations of plasma creatine, intramuscular creatine and their gradient with all‐cause mortality within a large population‐based cohort. Based on the literature, we hypothesise that higher plasma creatine is associated with a higher risk of mortality, whereas we expect higher intramuscular creatine to be associated with a lower risk of mortality. Directly measuring intramuscular creatine through muscle biopsies provides valuable insights, but is invasive and unfeasible for large‐scale studies. As a convenient alternative, intramuscular creatine can be estimated by dividing the total creatine pool by measured muscle mass as assessed with bioelectrical impedance analyses (BIA), as roughly 95% of the total creatine pool in the body is stored in skeletal muscle.^7^, ^8^, ^9^, ^10^, ^11^ Since 1.7% of the creatine pool is degraded to creatinine daily and excreted via the kidneys,^1^ the creatine pool can conveniently be assessed using 24‐hour urinary creatinine excretion. In our research, we conducted all analyses stratified by sex, as the creatine transporter gene is located on the X‐chromosome and previous studies have indicated sex‐based differences in creatine homeostasis and their associations with health outcomes.^5^, ^6^, ^12^, ^13^ This approach is in line with editorial calls for sex‐stratified reporting in medical research.^14^, ^15^

## 2 METHODS

### 2.1 Population

This concerns an observational study performed with data from the prospective Prevention of Renal and Vascular Endstage Disease (PREVEND). The PREVEND study was previously described in detail elsewhere.^16^, ^17^ The PREVEND study was approved by the medical ethics committee of the University Medical Center Groningen (METC96/01/022) and was carried out in accordance with the Declaration of Helsinki. All participants provided written informed consent. Our study was conducted in accordance with the STROBE (Strengthening the Reporting of Observational Studies in Epidemiology) guidelines.^18^ In short, the PREVEND study prospectively investigated risk factors for microalbuminuria and its relation to cardiovascular and renal disease in otherwise healthy adults.

85,421 inhabitants of the city of Groningen, The Netherlands, aged 28–75 years, were invited to participate in the study. In the 40,856 respondents, the urinary albumin concentration (UAC) was determined. Respondents were divided into a group with UAC <10 mg/L and a group with UAC ≥10 mg/L. Respondents with insulin‐dependent diabetes mellitus or pregnancy were considered ineligible and were excluded. 7768 eligible respondents with UAC ≥10 mg/L together with a random selection of 3395 participants with UAC <10 mg/L were asked for further participation in the study, of whom 2571 declined, resulting in a cohort of 8592 study participants in the first examination round of the study in 1997 to 1998. A second examination round took place from 2001 to 2003, encompassing 6894 participants and was considered the ‘baseline’ for the current study. We excluded 1724 participants with no data on plasma creatine or estimated intramuscular creatine, leading to a total of 5127 participants for the current study. Information on the flow of participants through the study is provided in Figure S1.

### 2.2 Main variable assessment

Both examination rounds comprised two visits to an outpatient clinic separated by 3 weeks. Baseline EDTA plasma samples were drawn between 8:00 and 10:00 a.m. from all participants, and aliquots of these samples were immediately stockpiled at −80°C until analysis.

Creatine was measured using a Vantera® NMR Clinical Analyser (LabCorp, Morrisville, NC).^19^ We performed creatine measurements in EDTA plasma samples of participants who had been instructed to perform an overnight fast. This minimalises the potential influence of alimentary creatine ingested with fish or meat on circulating creatine concentrations.^20^ Plasma samples were mixed (3:1 v/v) with citrate/phosphate buffer to adjust the pH to 5.3. This was necessary to separate the creatine and creatinine peaks, which overlap at physiological pH, to allow accurate quantification. Proton NMR spectra were acquired as previously described.^21^ Creatine results (*n* = 44) from the NMR quantification software agree well with routine enzymatic (creatinase)/spectrophotometry assay (*R* ^2^ = .995, slope = .99, intercept = 12.2). The coefficient of variation for intra‐ and inter‐assay precision for the NMR assay was 4.0%–4.9%.

Participants collected two consecutive 24‐h urine specimens after thorough oral and written instruction. During collection, participants were asked to refrain from heavy exercise and instructed to postpone urine collection in case of urinary tract infection, menstruation, or fever. Collected urine was stored cold (4°C) for a maximum of 4 days before the second visit. Specimens of the urine collections were stockpiled at −20°C until analysis. Urinary creatinine was measured by dry chemistry (Eastman Kodak, Rochester, USA). Intra‐ and inter‐assay coefficients of variation were .9% and 2.9%, respectively. The creatinine excretion rate (CER) was calculated as urinary creatinine concentration multiplied by 24‐h urine volume. The total creatine pool was defined as the 24‐h urinary creatinine excretion (mmol/day) divided by .017, given that 1.7% of the creatine pool is degraded to creatinine and excreted in the urine on a daily basis.^1^, ^22^

A single frequency BIA device (BIA 101, RJL systems, Akern SRL, Italy) was used to measure whole‐body electrical impedance at 50 kHz between the hand and the foot. Muscle mass was estimated using the equation by Janssen et al. 2000, which uses height, sex, age, resistance and reactance to estimate total skeletal muscle mass, rather than appendicular skeletal muscle mass, as assessed in many other equations.^23^ The estimated intramuscular creatine was defined as the creatine pool divided by the total skeletal muscle mass. For the primary analyses, we used muscle mass estimated with the equation of Janssen et al. 2000.^23^

The creatine gradient was defined as the estimated intramuscular creatine divided by the plasma creatine, reflecting the transcellular gradient for creatine.

### 2.3 Mortality

Data on all‐cause mortality were received from the Dutch Central Bureau for Statistics. Follow‐up time was defined as the period between the second examination round (baseline) and events defined as death, loss to follow‐up or the end of follow up time (01‐01‐2017), whichever came first. If a person had moved to an unknown destination, the date on which the person was dropped from the municipal registry was used as the census date.

### 2.4 Covariable assessment

Self‐administered questionnaires concerning demographics, cardiovascular and renal disease history, smoking habits and medication use were filled out by all participants prior to the first visit. No data about creatine supplementation were available. Height, weight and blood pressure were measured according to protocol.^17^ Body mass index (BMI) was calculated by dividing weight in kilograms by height, in meters, squared. Total cholesterol, HDL‐cholesterol, LDL‐cholesterol, triglycerides, glucose, albumin, urea, creatinine and cystatin C were measured using standard protocols, which have been described previously.^24^, ^25^, ^26^, ^27^ The estimated glomerular filtration rate (eGFR) was calculated using the 2012 CKD‐EPI equation using cystatin C, since creatinine‐based eGFR estimation is influenced by the total creatine pool. Protein intake was estimated using the Maroni formula.^28^

### 2.5 Statistical analyses

All statistical analyses were performed with R Studio statistical software (version 4.4.0). Normally distributed variables were presented as mean ± standard deviation (SD), skewed variables as median [interquartile range] and categorical data as number (valid percentage). Normality of distributions was assessed by visual inspection of histograms and Q‐Q plots. For all analyses, a two‐sided *p* value <.05 was considered statistically significant. All data presented and all analyses were stratified for sex.

Differences between males and females in baseline characteristics were tested using independent sample *t* test, Wilcoxon Mann–Whitney *U* test or chi‐squared test. Overlapping dot and box‐plots were used to present the distribution of intramuscular creatine, plasma creatine and creatine gradient, using the quasirandom geom function in R to offset points within categories to reduce overplotting.

Potential determinants for plasma creatine and intramuscular creatine in males and females were assessed using multivariable linear regression analyses, adjusting for age, sex, BMI, eGFR and urinary albumin excretion. Coefficients were presented as standardized betas, referring to the number of standard deviations a dependent variable changes per standard deviation increase of the independent variable.

Associations of plasma creatine, intramuscular creatine and the creatine gradient with all‐cause mortality in the whole population, and females and males separately, were assessed using Cox proportional hazard models. Hazard ratios were computed per doubling. The proportional hazards assumption was verified visually with plots of the scaled Schoenfeld residuals and was not violated in any of the models. Cox regression analyses were adjusted for a priori selected potential confounders, including age, sex, BMI, eGFR, urinary albumin excretion, smoking status, alcohol intake, history of CV disease, triglycerides, HDL cholesterol, LDL cholesterol, hypertension and diabetes. In sensitivity analyses, we investigated associations with mortality of intramuscular creatine based on different equations for muscle mass (equations of Sergi et al. 2015 and Kyle et al. 2003^29^, ^30^), intramuscular creatine without high and low outliers, intramuscular creatine as measured in the first examination round of the study, and intramuscular creatine with additional adjustment for protein intake and for appendicular skeletal muscle mass index (ASMI, calculated with the formula of by Sergi et al. 2015^29^ as recommended by the European consensus on definition and diagnosis of Sarcopenia^31^). To visualise the continuous associations of estimated intramuscular creatine with all‐cause mortality, the continuous variable was plotted against the risk of all‐cause mortality, using the median value as the reference point. To account for potential bias that could result from the exclusion of participants with missing values, multiple imputation using Fully Conditional Specification was performed using the ‘mice’ package to obtain five imputed data sets. The algorithm was run for 30 iterations and convergence of the Markov chains was evaluated with trace plots of the mean and variance. To confirm that imputed values were biologically plausible, the distributions of the imputed values were visually investigated and compared to the distribution of the observed values. Analyses were performed in each of the data sets and results were pooled using Rubin's rules.^32^ Apart from the baseline table and unless otherwise stated, analyses were performed using imputed data sets.

## 3 RESULTS

### 3.1 Baseline characteristics

**FIGURE 1:**
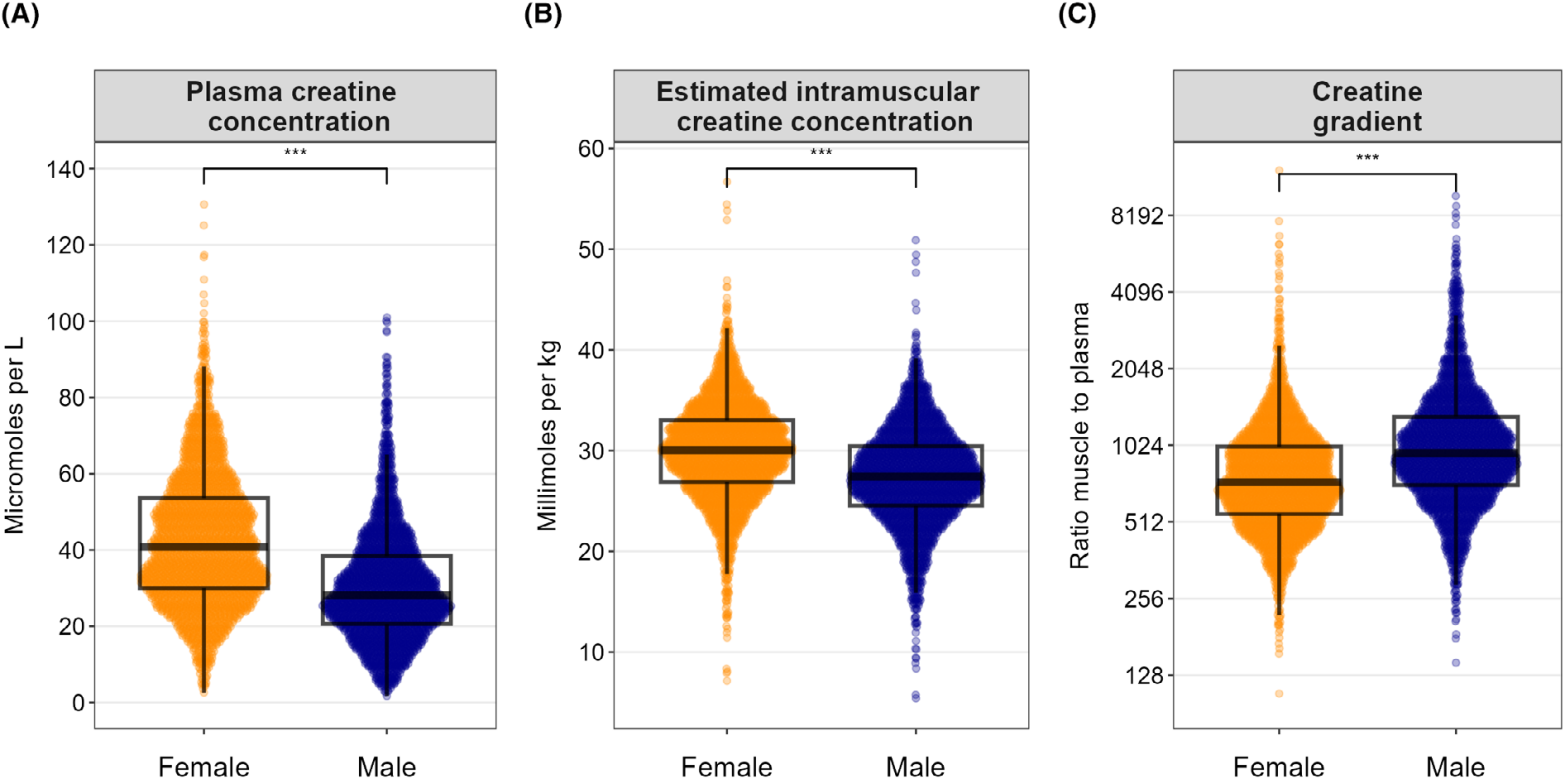
Combined dot plot and box plot demonstrating the distributions of plasma creatine concentration (females: 41 [30–54] μmol/L, males: 28 [21–38] μmol/L), estimated intramuscular creatine concentration (females: 30.0 ± 5.0 μmol/kg, males: 27.4 ± 5.0 μmol/kg) and the creatine gradient (females: 734 [550–1011], males: 955 [715–1324]), according to sex. The quasirandom geom function in R was used to offset points within categories to reduce overplotting. The creatine gradient was defined as the estimated intramuscular creatine divided by the plasma creatine, reflecting the transcellular gradient for creatine.

An overview of baseline characteristics of the 5170 participants with a mean age of 54 ± 12 years, a mean BMI of 26.7 ± 4.4 kg/m^2^ and a mean eGFR of 91 ± 20 mL/min/1.73 m^2^, is shown in Table 1. Plasma creatine and intramuscular creatine were higher in females compared to males (both *p* < .001); whereas the creatine gradient was higher in males compared to females (*p* < .001) (see Figure 1).

**TABLE 1:** Baseline characteristics of the population‐based cohort.

Variables	Total cohort (*n* = 5170)	Females (*n* = 2669)	Males (*n* = 2501)	*p*‐Value
Plasma creatine, μmol/L	34 [25–47]	41 [30–54]	28 [21–38]	<.001
Intramuscular creatine^a^, mmol/kg	28.7 ± 5.2	30.0 ± 5.0	27.4 ± 5.0	<.001
Creatine gradient (intramuscular: plasma)	843 [613–1158]	734 [550–1011]	955 [715–1324]	<.001
Age, years	54 ± 12	53 ± 12	55 ± 12	<.001
BMI, kg/m^2^	26.7 ± 4.4	26.6 ± 4.9	26.8 ± 3.8	.07
eGFR, mL/min/1.73 m^2^	91 ± 20	91 ± 19	90 ± 21	.11
Urinary albumin excretion	9 [6–16]	8 [6–13]	10 [7–21]	<.001
Smoking, *n* (%) current	1435 (28)	754 (28)	681 (27)	.47
Alcohol, *n* (%) daily consumption	1326 (26)	503 (19)	823 (33)	<.001
Cardiovascular history, *n* (%)	317 (6)	91 (3)	226 (9)	<.001
Triglycerides	1.1 [.8–1.6]	.9 [.7–1.4]	1.3 [.9–1.8]	<.001
HDL‐cholesterol	1.3 ± .3	1.5 ± .3	1.2 ± .3	<.001
LDL‐cholesterol	3.0 ± .8	3.0 ± .8	3.0 ± .7	.53
Hypertension, *n* (%)	1703 (33)	769 (29)	934 (37)	<.001
Diabetes, *n* (%)	308 (6)	134 (5)	174 (7)	.004

^a^ Refers to the estimated intramuscular creatine concentration, calculated as the creatine pool divided by total muscle mass.

### 3.2 Determinants of Creatine Concentrations

**FIGURE 2:**
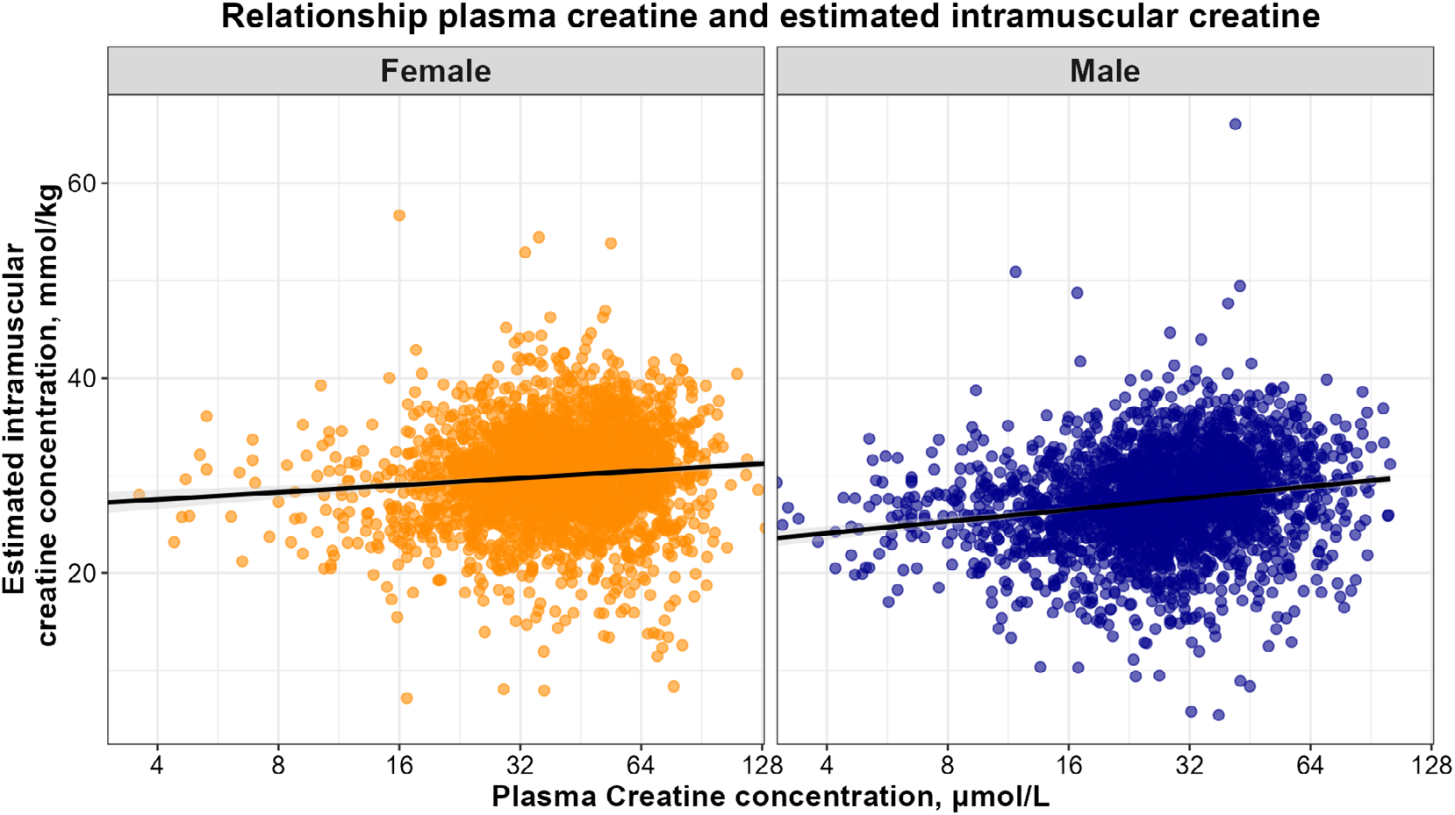
Dot plot of estimated intramuscular creatine concentration and plasma creatine concentration. In females the standardized beta was .10 (.06; .14), with an *R* ^2^ of <.01. In males the standardized beta was .12 (.08; .17), with an *R* ^2^ of .03.

An overview of linear regression analyses is shown in Table 2. Plasma creatine and intramuscular creatine were positively associated, with similar strengths observed between sexes (.10 (.06; .14), *p* < .001 and .12 (.08; .17), *p* < .001 for females and males, respectively), as shown in Figure 2. In both sexes, age was positively associated with plasma creatine and negatively associated with intramuscular creatine. In both sexes, BMI, eGFR and albuminuria were all positively associated with both plasma creatine and intramuscular creatine. The positive associations with plasma creatine were stronger, resulting in negative associations with creatine gradient. Notably, albuminuria was not associated with creatine gradient in females.

**TABLE 2:** Linear regression analyses.

Independent variable ↓	Dependent variable ➔	Intramuscular creatine concentration^a^		Plasma creatine		Creatine gradient	
		Std beta (95% CI)	*p*‐Value	Std beta (95% CI)	*p*‐Value	Std beta (95% CI)	*p*‐Value
Intramuscular creatine concentration^a^	All	–	–	.11 (.08; .13)	<.001	.26 (.22; .30)	<.001
	Female	–	–	.08 (.05; .12)	<.001	.28 (.25; .32)	<.001
	Male	–	–	.11 (.08; .15)	<.001	.26 (.22; .29)	<.001
Plasma creatine	All	.12 (.09; .15)	<.001	–	–	−.96 (−.97; −.95)	<.001
	Female	.10 (.06; .14)	<.001	–	–	−.97 (−.98; −.96)	<.001
	Male	.12 (.08; .17)	<.001	–	–	−.96 (−.97; −.94)	<.001
Creatine gradient^b^	All	.28 (.25; .31)	<.001	−.89 (−.90; −.88)	<.001	–	–
	Female	.31 (.27; .35)	<.001	−.88 (−.89; −.87)	<.001	–	–
	Male	.26 (.22; .30)	<.001	−.90 (−.91; −.88)	<.001	–	–
Age	All	−.10 (−.14; −.07)	<.001	.23 (.20; .26)	<.001	−.27 (−.30; −.23)	<.001
	Female	−.14 (−.18; −.09)	<.001	.22 (.18; .27)	<.001	−.28 (−.32; −.23)	<.001
	Male	−.06 (−.11; −.01)	.02	.24 (.19; .29)	<.001	−.26 (−.31; −.21)	<.001
Sex	All	−.50 (−.55; −.45)	<.001	−.76 (−.81; −.72)	<.001	.58 (.53; .64)	<.001
	Female	–	–	–	–	–	–
	Male	–	–	–	–	–	–
BMI	All	.10 (.07; .13)	<.001	.18 (.15; .20)	<.001	−.14 (−.17; −.18)	<.001
	Female	.04 (.01; .08)	.008	.12 (.09; .15)	<.001	−.10 (−.10; −.14)	<.001
	Male	.20 (.15; .25)	<.001	.28 (.24; .33)	<.001	−.21 (−.26; −.16)	<.001
eGFR	All	.15 (.11; .18)	<.001	.18 (.15; .21)	<.001	−.17 (−.09; −.03)	<.001
	Female	.14 (.09; .18)	<.001	.15 (.11; .20)	<.001	−.14 (−.19; −.10)	<.001
	Male	.15 (.06; .14)	<.001	.19 (.15; .24)	<.001	−.18 (−.23; −.13)	<.001
Albuminuria	All	.11 (.08; .14)	<.001	.07 (.05; .10)	<.001	−.03 (−.06; −.01)	.02
	Female	.10 (.06; .15)	<.001	.05 (.01; .08)	.02	−.01 (−.05; .03)	.70
	Male	.10 (.06; .13)	<.001	.08 (.04; .11)	<.001	−.04 (−.08; −.01)	.04

*Note*: The models are adjusted for age, sex, body mass index, eGFR_cys_ and urinary albumin excretion.

^a^ Refers to the estimated intramuscular creatine concentration, calculated as the creatine pool divided by total muscle mass.

^b^ Refers to the intramuscular: plasma creatine ratio.

### 3.3 All‐cause mortality

During a median follow‐up of 10.9 [8.4–13.4] years, 748 (14%) of the participants died.

**FIGURE 3:**
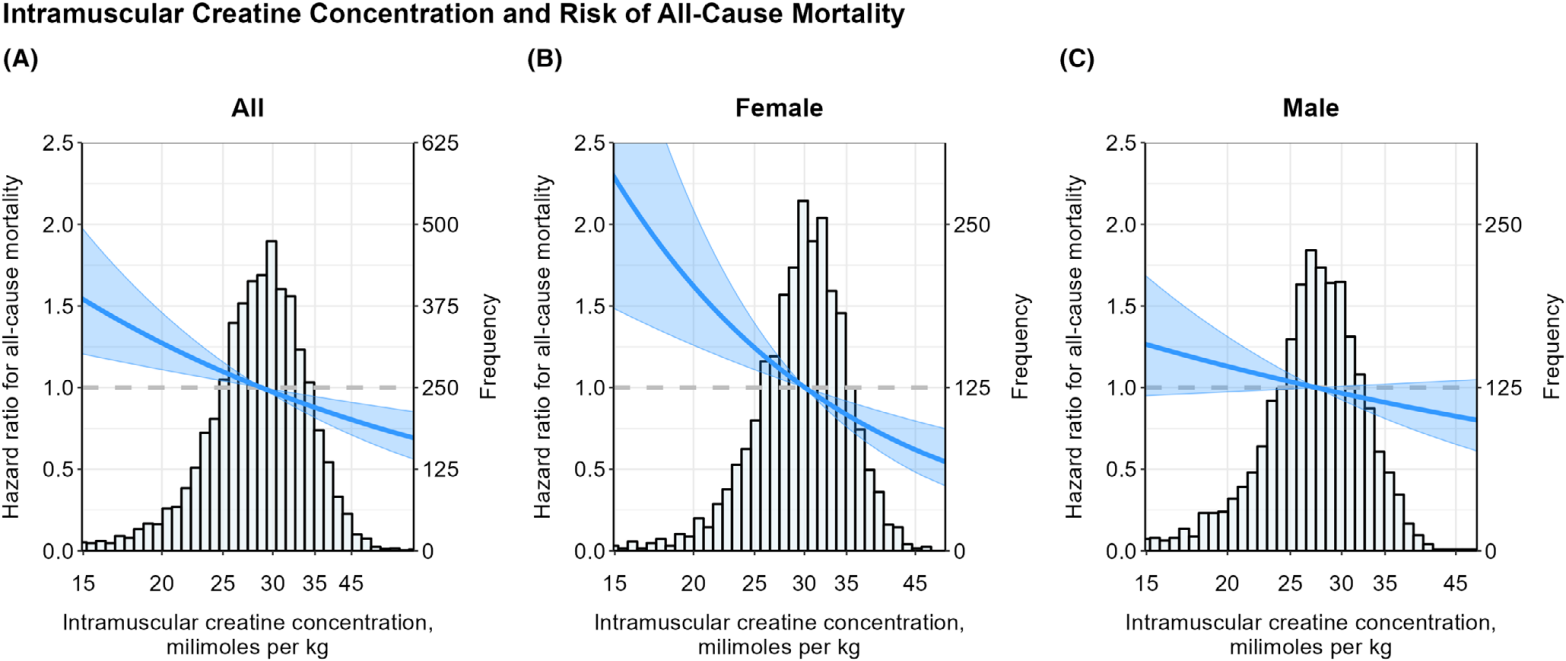
Visual representation of the association of the estimated intramuscular creatine concentration with all‐cause mortality according to sex. Analyses are adjusted for age, sex, body mass index, eGFR, urinary albumin excretion, smoking, alcohol and cardiovascular history, triglycerides, HDL‐cholesterol, LDL‐cholesterol, hypertension and diabetes.

An overview of Cox regression analyses is shown in Table 3. A higher intramuscular creatine was associated with a lower risk of all‐cause mortality (HR (95% CI): .52 (.40; .68) per doubling; *p* < .001) after adjusting for basic confounders. This association was primarily driven by the effect in females, as indicated by the difference in point estimates (HR (95% CI): .37 (.25; .58); *p* < .001 in females versus HR (95% CI): .62 (.45; .87); *p* = .005 in males). After further adjustment for potential confounders, the association remained significant in the overall population and in females but was lost in males. A graphical overview of the associations of estimated intramuscular creatine with mortality is shown in Figure 3.

**TABLE 3:** Cox regression analyses of plasma creatine, intramuscular creatine and creatine gradient with all‐cause mortality.

	Model 1		Model 2		Model 3	
	HR (95% CI)	*p*‐Value	HR (95% CI)	*p*‐Value	HR (95% CI)	*p*‐Value
Plasma creatine						
All	1.04 (.94; 1.16)	.48	1.01 (.90; 1.12)	.92	1.04 (.93; 1.15)	.56
Female	1.00 (.82; 1.23)	.93	1.01 (.83; 1.23)	.91	1.02 (.84; 1.25)	.82
Male	1.06 (.94; 1.20)	.36	1.02 (.90; 1.17)	.72	1.06 (.93; 1.21)	.39
Intramuscular creatine concentration^a^						
All	.52 (.40; .68)	<.001	.61 (.47; .79)	<.001	.62 (.48; .79)	<.001
Female	.37 (.25; .58)	<.001	.42 (.27; .65)	<.001	.43 (.28; .66)	<.001
Male	.62 (.45; .87)	.005	.73 (.52; 1.02)	.06	.73 (.53; 1.02)	.07
Creatine gradient^b^						
All	.88 (.80; .97)	.01	.91 (.83; 1.02)	.11	.90 (.81; 1.00)	.05
Female	.83 (.69; 1.02)	.07	.84 (.69; 1.03)	.09	.84 (.69; 1.03)	.09
Male	.89 (.79; 1.01)	.06	.93 (.82; 1.06)	.26	.91 (.80; 1.03)	.14

*Note*: All HR are presented per doubling of the variable. Model 1: Adjusted for age, sex, body mass index, eGFR, urinary albumin excretion. Model 2: As model 1, additionally adjusted for smoking, alcohol and cardiovascular history. Model 3: As model 2, additionally adjusted for triglycerides, HDL‐cholesterol, LDL‐cholesterol, hypertension and diabetes.

^a^ Refers to the estimated intramuscular creatine concentration, calculated as the creatine pool divided by total muscle mass. ^b^ Refers to the intramuscular: plasma creatine ratio.

A higher plasma creatine was not associated with all‐cause mortality, neither in the whole population, nor in females or males. A higher plasma creatine gradient was associated with a lower risk of all‐cause mortality (HR (95% CI): .88 (.80; .97); *p* = .013) after adjusting for potential confounders. However, this association lost significance after further adjusting for additional potential confounders.

To assess the robustness of the association of estimated intramuscular creatine with mortality, we performed various sensitivity analyses, which are shown in Table 4. These analyses demonstrate that the association of intramuscular creatine with mortality remained significant regardless of the equation used to estimate muscle mass, regardless of whether data from the first or second visit were used for creatine pool assessment, regardless of low or high outliers, and regardless of additional adjustment for protein intake or ASMI.

**TABLE 4:** Sensitivity analyses of the cox regression analyses of intramuscular creatine concentration using various equations.

	HR (95% CI)	*p*‐Value
Intramuscular creatine^a^ estimated using muscle mass via BIA with Janssen equation (default)		
All	.62 (.48; .79)	<.001
Female	.43 (.28; .66)	<.001
Male	.73 (.53; 1.02)	.07
Intramuscular creatine^a^ estimated using muscle mass via BIA with Kyle equation		
All	.62 (.47; .82)	<.001
Female	.48 (.30; .75)	.002
Male	.71 (.51; 1.01)	.05
Intramuscular creatine^a^ estimated using muscle mass via BIA with Sergi equation		
All	.62 (.47; .81)	<.001
Female	.49 (.31; .78)	.002
Male	.70 (.49; .98)	.04
Intramuscular creatine^a^ estimated using only creatine pool on first visit		
All	.74 (.60; .92)	.006
Female	.52 (.37; .75)	.004
Male	.89 (.67; 1.17)	.39
Intramuscular creatine^a^ estimated using only creatine pool on second visit		
All	.75 (.60; .92)	.007
Female	.52 (.37; .75)	<.001
Male	.90 (.678; 1.18)	.44
Intramuscular creatine^a^ without low outliers^b^		
All	.69 (.52; .91)	.008
Female	.50 (.31; .81)	.005
Male	.81 (.57; 1.14)	.22
Intramuscular creatine^a^ without high outliers^b^		
All	.62 (.48; .82)	<.001
Female	.45 (.29; .69)	<.001
Male	.76 (.54; 1.06)	.11
Intramuscular creatine^a^, additionally adjusted for protein intake		
All	.63 (.47; .85)	.002
Female	.42 (.26; .69)	<.001
Male	.78 (.54; 1.23)	.18
Intramuscular creatine^a^, additionally adjusted for ASMI		
All	.58 (.45; .75)	<.001
Female	.43 (.28; .65)	<.001
Male	.67 (.48; .93)	.02

*Note*: All HR are presented per doubling of the variable. The analyses are adjusted for age, sex, body mass index, eGFR, urinary albumin excretion, smoking, alcohol and cardiovascular history, triglycerides, HDL‐cholesterol, LDL‐cholesterol, hypertension and diabetes.

^a^ Refers to the estimated intramuscular creatine concentration, calculated as the creatine pool divided by total muscle mass.

^b^ Outliers were defined as values higher or lower than 3 standard deviations from the mean. 23 outliers were excluded for the analyses without the lowest outliers, and 27 for the analyses without the highest outliers.

## 4 DISCUSSION

The key finding of our study was that higher estimated intramuscular creatine concentration was strongly and independently associated with lower all‐cause mortality in a large, population‐based cohort, which was in line with our hypothesis. Interestingly, this association was more pronounced in females, where the association was much stronger compared to males. Both plasma creatine and intramuscular creatine concentrations were higher in females, whereas the transcellular concentration gradient (intramuscular: plasma) was higher in males. Notably, plasma creatine did not reflect intramuscular creatine stores.

The observation that plasma creatine was higher in females compared to males aligns with findings in earlier studies.^5^, ^6^, ^13^, ^33^, ^34^ Besides female sex, eGFR, age and BMI were also positively associated with plasma creatine, consistent with existing literature.^5^, ^13^, ^35^ Intramuscular creatine concentration was ~9% higher in females compared to males. This finding is similar to biopsy‐based data from Forsberg et al. (1991), showing a ~10% higher creatine concentration in females compared to males.^36^ This difference was not explained by the sex difference in muscle fibre composition: as males tend to have a higher percentage of fast twitch muscle fibres,^37^, ^38^ which have a higher creatine content compared to slow twitch muscle fibres,^39^ the contrary would be expected. In contrast with our study, Machek et al. (2020) found no sex difference in muscle creatine content, muscle creatine transporters, serum creatine and serum creatinine in powerlifters and sedentary controls.^40^

Although plasma creatine and intramuscular creatine were correlated in our study, only a small proportion (in females <1% and in males 3%) of the inter‐individual variability in plasma creatine was explained by intramuscular creatine. Hence, plasma creatine does not reflect intramuscular concentrations. The plasma creatine concentration rather is a consequence of a variety of different mechanisms, including creatine intake, endogenous creatine synthesis, renal tubular reabsorption of creatine, expression and activity of the creatine transporter for intracellular creatine uptake, cellular creatine release resulting from cell damage and creatine release transporters for rapid equilibration of the intracellular creatine pool.^41^ The higher creatine gradient in men suggests a higher capacity to accumulate creatine intracellularly against a large concentration gradient. Although the creatine transporter is thought to be the main determinant of intracellular creatine concentrations and the transcellular gradient,^1^ a previous study found similar amounts of creatine transporter between sexes.^40^

Contrary to our hypothesis, and despite associations of plasma creatine with a variety of unfavourable metabolic and cardiovascular markers,^5^, ^6^ we found no association of plasma creatine with mortality in the present study. On the other hand, in line with our hypothesis, higher intramuscular creatine was associated with lower mortality, independently of a wide variety of cardiovascular risk factors and other potential confounders including protein intake. To our knowledge, this study is the first to describe this association. Previous studies already linked different markers indicating a small bodily creatine pool size with higher mortality across various populations (e.g. low serum creatinine concentrations in patients admitted to the intensive care unit,^42^, ^43^ low creatine index in patients receiving haemodialysis,^44^ low urinary creatinine excretion in kidney transplant recipients,^45^, ^46^ and low plasma creatinine concentrations adjusted for cystatin C concentration and urinary creatinine excretion in the currently investigated population‐based PREVEND cohort and the NHANES cohort^47^, ^48^). Notably, these creatine status markers all reflect absolute creatine pool size rather than intramuscular concentrations of creatine, as investigated in our study.

Urinary creatinine excretion, directly reflecting total creatine pool size, is commonly used as a surrogate marker for muscle mass. Our findings demonstrate that muscle mass and creatine pool size are in fact distinct metrics with independent importance, as their ratio (intramuscular creatine concentration) is associated with outcomes. Importantly, intramuscular creatine concentration remained strongly associated with mortality after adjusting for muscle mass assessed using the appendicular skeletal muscle mass index. Muscle mass itself may protect against mortality due to a variety of mechanisms including its role as a protein and energy reserve during catabolic stress,^49^ contribution to fall prevention and reduced frailty,^50^ improved insulin sensitivity,^51^ reduction of systemic inflammation^52^ and enhanced cardiovascular health.^53^ A higher intramuscular creatine concentration may reflect better muscle quality or function and an overall higher creatine status, with possibly an additional protective effect due to increased resilience against frailty,^54^, ^55^ improved neurocognitive function and neuroprotective properties,^55^, ^56^, ^57^ anti‐oxidant properties and minimised bone loss, especially in aging physiologically stressed populations.^55^

Borderline associations of the creatine gradient with mortality suggest there could be a protective effect of a larger transcellular creatine gradient. As creatine transporter expression is influenced by numerous factors, including both intracellular and extracellular creatine concentrations, dietary factors such as creatine supplementation or fasting, and hormonal factors,^1^ many potential pathways could be explored.

The sex differences in our observations, both in the observed concentrations as well as in associations with mortality, are remarkable. The association between intramuscular creatine and mortality remained robust in females after adjusting for a wide spectrum of potential confounders; whereas in males we observed a similar but weaker trend, which lost significance following adjustment. Potential explanations may involve hormonal regulation, sex‐specific differences in transporter expression or activity or differential dietary and metabolic factors influencing creatine homeostasis. Future research is needed to explain this finding.

A key strength of the study is the large, well‐characterised population‐based cohort with long‐term follow‐up, ensuring large statistical power, allowing for both overall and sex‐stratified analyses, and for adjustment for many potential confounders. Combining urinary creatinine excretion and BIA measurements allowed for a non‐invasive and accessible manner to estimate intramuscular creatine in such a large population. Although circulating creatine fluctuates over time, with peaks mainly following the ingestion of creatine‐rich meals,^20^ the effects of these fluctuations were minimised by obtaining blood samples after an overnight fast. Furthermore, extensive sensitivity analyses confirm the robustness of the observed association of estimated intramuscular creatine with mortality in females.

Some limitations of the study need to be addressed. While creatinine excretion reliably estimates the total creatine pool size, BIA provides equation‐based estimates of muscle mass rather than direct measurements and is influenced by hydration status, fat distribution and the chosen equations. This adds some uncertainty to intramuscular creatine estimates, which we mitigated through sensitivity analyses using alternative muscle mass equations. Although our methods present an elegant way to non‐invasively estimate intramuscular creatine in a large cohort, it is based on the assumption that the largest proportion of bodily creatine is present in muscle tissue.^7^, ^8^, ^9^ This does not take into account any possible variability in the bodily distribution of creatine between individuals or sexes. Furthermore, due to the observational nature of our study, we cannot establish causality; the possibility of residual confounding cannot be excluded.

In conclusion, our study shows that higher estimated intramuscular creatine was strongly associated with lower all‐cause mortality in females, whereas only a weaker trend was observed in males. Future research should elucidate causality and potential mechanisms, as well as further explore the remarkable sex difference in our observations.

## AUTHOR CONTRIBUTIONS

Conception of the study: CSED, AP, SJLB. Laboratory analyses: MAC. Data analyses: AP. Writing of the manuscript: CSED, AP. Critical review of the manuscript: all authors.

## FUNDING INFORMATION

The Dutch Kidney Foundation supported the infrastructure of the PREVEND program from 1997 to 2003 (Grant E.033). The University Medical Center Groningen supported the infrastructure from 2003 to 2006. Dade Behring, Ausam, Roche and Abbott financed laboratory equipment and reagents. The Dutch Heart Foundation supported studies on lipid metabolism from 2001 to 2005 (Grant 2001–005). The funders had no role in the conception, design, or preparation of this manuscript.

## CONFLICT OF INTEREST STATEMENT

The UMCG received funding from Crearene AG, Switzerland, for another study on the topic of creatine. M.A. Connely is an employee of Labcorp and holds stock in Labcorp.

## CONSENT

Written informed consent for participation in the study was obtained from all participants.

## Supporting information


Figure S1.

## Data Availability

Data described in the manuscript, code book and analytic code will be made available upon reasonable request.
