# Supplementary figures and images for: Plasma creatine, estimated intramuscular creatine, transcellular gradient and the risk of mortality: Results from the PREVEND study
Source: Eur J Clin Invest. 2025 Aug 21;56(1):e70110. doi: 10.1111/eci.70110 (PMC12811838; doi:10.1111/eci.70110)

# Supplementary material


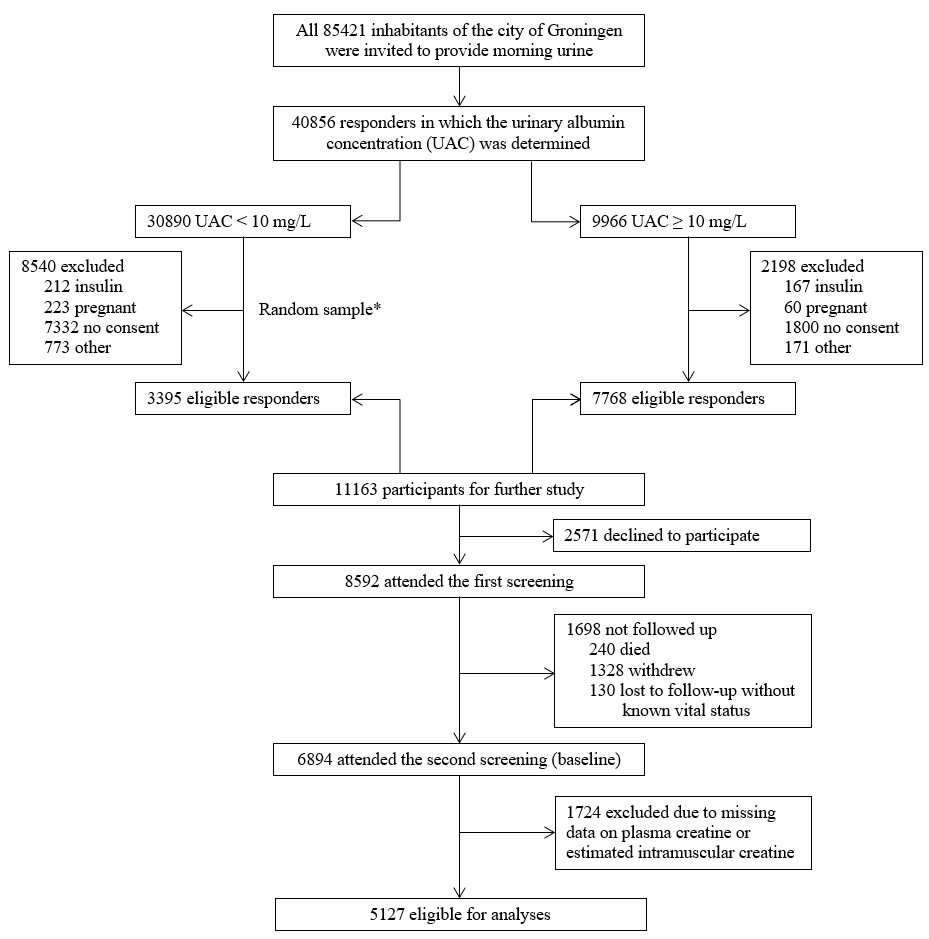


**Figure S1.** Participant flow through the study.

Supplement: Supplementary file 1 — Figure S1. [file ECI-56-e70110-s001.docx]
